# A lightweight method for maize seed defects identification based on Convolutional Block Attention Module

**DOI:** 10.3389/fpls.2023.1153226

**Published:** 2023-09-05

**Authors:** Chao Li, Zhenyu Chen, Weipeng Jing, Xiaoqiang Wu, Yonghui Zhao

**Affiliations:** ^1^ College of Computer and Control Engineering, Northeast Forestry University, Harbin, China; ^2^ School of Mechanical Engineering, Inner Mongolia University for Nationalities, Tongliao, Inner Mongolia Autonomous Region, China

**Keywords:** MobileNetv3-Large, image classification, transfer learning, CBAM, lightweight network

## Abstract

Maize is widely cultivated and planted all over the world, which is one of the main food resources. Accurately identifying the defect of maize seeds is of great significance in both food safety and agricultural production. In recent years, methods based on deep learning have performed well in image processing, but their potential in the identification of maize seed defects has not been fully realized. Therefore, in this paper, a lightweight and effective network for maize seed defect identification is proposed. In the proposed network, the Convolutional Block Attention Module (CBAM) was integrated into the pretrained MobileNetv3 network for extracting important features in the channel and spatial domain. In this way, the network can be focused on useful feature information, and making it easier to converge. To verify the effectiveness of the proposed network, a total of 12784 images was collected, and 7 defect types were defined. Compared with other popular pretrained models, the proposed network converges with the least number of iterations and achieves the true positive rate is 93.14% and the false positive rate is 1.14%.

## Introduction

Maize is an important feed crop in animal husbandry and aquaculture industry, as well as one of the essential raw materials in food industry. Rapid and accurate identification of maize seed varieties not only has important application value in maize planting, but also can effectively avoid the occurrence of adverse phenomena such as mixed seed and mixed processing. In maize automatic sorting system, the industrial camera needs to collect images of the seeds, so that we can screen and compare whether the appearance of the seeds is complete and normal, and the final classification accuracy of the sorting system is highly dependent on the performance of maize image classification algorithm.

Traditional methods (Hinton et al., 2006; Kiratiratanapruk and Sinthupinyo, 2011; Huang et al., 2019) for the classification of maize seed images generally pre-process the images first, then extract the shape, color and other feature information of each maize image by artificial means, screen and integrate the extracted features. Using professional industrial machines to extract maize seeds can not only avoid the influence of other light sources, but also achieve automatic acquisition to improve the collection efficiency. Previous study has proposed several classification algorithm by deep learning. (Krizhevsky et al., 2017) proposed the convolutional neural network AlexNet, which won the first place in the ImageNet competition, and reduced the Top-5 classification error rate of 1000 types of images to 10%. (Xu et al., 2021) established a variety classification model by using multi-layer perceptron, the overall classification accuracy reached more than 95%. (Alotaibi and Alotaibi, 2020) used the residual network ResNet to classify hyperspectral images. The accuracy was 95.33% on the Salinas dataset and 90.57% on the Indian Pines dataset. (Cui et al., 2019) uses the improved VGG16 to classify the four types of images, and replaces the SoftMax classifier in the VGG16 network with the 4-label SoftMax classifier. The final test accuracy of the model is 95%. EfficientNet (Soleimanipour et al., 2022) was used to classify the four pistachio varieties, based on Python programming the Kears API and TensorFlow machine learning framework. The results show that, the average accuracy rate and recall rate of this model are 96.73% and 96.70%, respectively. Compared with traditional machine learning, using deep learning to solve the problem of image classification can not only avoid the influence of human factors, but also greatly improve the recognition accuracy and have stronger observability.

The application of deep learning in agricultural production has a significant impact as it can improve crop productivity and quality. Taking bananas and apples as examples, (Wu et al., 2021; Fan et al., 2022b; Wu et al., 2022) proposed deep learning-based methods for defect detection in bananas and apples, respectively. These methods can quickly and accurately detect defects in fruits and achieve real-time detection, providing farmers with more efficient means of fruit screening and quality control.

For the overall recognition and localization of banana clusters, (Wu et al., 2023) proposed a deep learning based method with an accuracy rate of 93.2%. Using the YOLOv5-B model for banana localization and detection, the average processing time per image is only 9 ms. These research results can help farmers automatically identify and count the number of banana clusters during the growth and harvesting process, improving production management efficiency and accuracy.

In apple production, (Fan et al., 2022b) proposed a real-time defect detection method based on the YOLOv4 network. The average detection accuracy in online testing reached 93.9%, and it can evaluate 4 fruits per second. This method can assist farmers in quickly and accurately screening out defective apples from a large quantity, improving apple quality and market competitiveness.

In addition, (Wang et al., 2022) proposed a maize defect detection method based on the watershed algorithm, providing a new solution for maize production. This method can effectively identify good seeds and bad seeds, with an average accuracy rate of 95.63% and an average recall rate of 95.29%. It can help farmers quickly select high-quality seeds during the planting process, improving planting success rate and yield.

In conclusion, the application of deep learning methods in agricultural production plays a crucial role. Through automated image recognition and defect detection techniques, it can enhance the quality and productivity of agricultural products, provide farmers with more accurate and efficient production management tools, and make a significant contribution to promoting agricultural development.

In this paper, our main contributions are as follows: Firstly, we propose an improved MobileNetv3 network, which can reduce model parameters and reduce real-time computation. The requirements for equipment resources are low and can be applied to mobile devices. Secondly, we introduce the Convolutional Block Attention Module to the MobileNetv3 network to improve the fitting ability and generalization ability of the network. Finally, we use our own maize seed dataset to test the new method, which is derived from the maize image in GrainSpace (Fan et al., 2022a), and has achieved more than 90% recognition accuracy. Experimental verification has demonstrated that the proposed algorithm in this paper is capable of classifying maize based on its appearance, and further detecting the types of defects present, thus achieving intelligent detection of maize defects and effectively reducing labor costs. This algorithm holds great potential for wide-ranging applications and promising future development.

## Related work

### Transfer learning

The transfer learning method can greatly save the time of training model and significantly reduce the hardware resources required by deep learning. For this reason, more and more scholars adopt transfer learning method to train their own data sets. (Xie et al., 2022) used Inceptionv3 and DenseNet201 as feature extractors at the same time, and used the dual transfer learning framework to reach the accuracy rate, recall rate and F1 values of 95.11%, 95.33%, and 95.15% on 10 types of seabird data sets. (Fraiwan et al., 2022) applied the transfer learning technology to the convolution neural network model to classify three maize diseases. The average accuracy rate reached 98.6%, which proved that transfer learning can greatly improve the accuracy of classification. (Xiang et al., 2019) applied lightweight network MobilleNetv2 to the task of fruit classification, and used Softmax as the classifier for feature classification. Finally, the accuracy rate was obtained 85.12%. (Das et al., 2023) applied the transfer learning to the DenseNet169 network to classify 11 kinds of garbage that frequently occur in outdoor environment, and finally achieved the accuracy of 93.10%. (Huang et al., 2017) put forward the method of transfer learning to solve the obstacles in SAR target classification. (Tammina, 2019) proposed to use the VGG16 model of transfer learning to classify cat and dog pictures, and achieved an accuracy rate of 95.40%, which is 16.2% higher than that without transfer learning. (Kaya et al., 2019) used DNN network of transfer learning to verify the influence of deep neural network on plant classification in four different transfer learning models. The experimental results show that transfer learning can optimize automatic plant identification and improve low-performance plant classification models.

### MobileNet

MobileNet network model is a lightweight network model proposed by Google. Compared with the traditional convolutional neural network, MobileNet has the advantages of fewer parameters and lower delay. At present, MobileNet series networks include MobileNetv1, MobileNetv2 (Huu et al., 2022; Młodzianowski, 2022) and MobileNetv3 (Howard et al., 2019; Hussain et al., 2021; Zhao and Wang, 2022; Zhao et al., 2022). MobileNetv3 is Google’s new invention after MobileNetv2, and its main improvement is to add SE-net after the deep separable convolution in MobileNetv2, which automatically obtains the importance of each feature channel by learning, and suppresses some feature information that is not useful for the current task. In addition, MobileNetv3 integrates the four characteristics of MobileNetv2 and MobileNetv1, which first uses the convolution of 1×1 for dimensionality upgrading, and introduces the inverse residual structure of MobileNetv2 linear bottleneck. Then, 3x3 depth separable convolution is performed to reduce the computational amount of the network. Then, through the lightweight SE-net attention model, the net-work pays attention to more useful channel information to ad-just the weight of each channel. Finally, the h-swish activation function is used instead of the swish function to reduce the amount of operation and improve performance. After changing the Bneck structure, compared with MobileNetv2, MobileNetv3 achieves the same accuracy on COCO, the speed is 25% faster, and the segmentation algorithm is also improved.

### Convolutional block attention module

The application of Attention mechanisms (Zhang et al., 2022) enables the neural network to focus on important features and suppress unnecessary features. This paper uses the Convolutional Block Attention Module (Woo et al., 2018). (Liang et al., 2022) used CNN to mine the deep features of gold price data and improved the feature extraction ability of the network through CBAM. The experiment proved that this method could improve the prediction accuracy and was superior to other models. (Ma et al., 2019) proposed CBAM-GAN generative adversarial network, which can significantly improve the quality of generated images. (Li et al., 2022) adopted MobileNetv3 to detect floating objects on the water surface and CBAM to enhance feature fusion. The experiment showed that the detection accuracy of the improved model increased by 2.9% and the detection speed increased by 55%. (Pei et al., 2022) used the YOLOv4 model with CBAM to detect weeds in the field, which reduced the total number of tags in 1000 images by half, and the average accuracy reached 86.89%, thus improving the efficiency of weed detection. (Chen et al., 2023) used the MobileNetv3-large network combined with CBAM for crack detection. The experiment showed that the model had better performance and the crack identification accuracy reached 99.69%. CBAM can improve the training efficiency and prediction accuracy of CNN, which is a combination of channel attention mechanism and spatial attention mechanism, which can be embedded in the module of CNN and conduct end-to-end training together with CNN, with only a small amount of computation added.

## Methods

### The method of MobileNetv3-Large

We used MobileNetv3-Large as the image classification model (Qian et al., 2021). MobileNetv3 introduces the depthwise convolution. The convolution kernel of the depthwise separable convolution is equal to the number of input channels, that is, a convolution kernel performs a convolution operation on a feature map of the previous layer separately to obtain the number of output channels equal to the number of input channels. Compared with conventional convolution, the number of parameters of the depthwise convolution can be saved by 1/3, and the number of layers of the neural network can be deeper under the premise of the same number of parameters, so that the model can be lightweight.

MobileNetv3 uses an inverted residual structure. It can effectively avoid the problem of gradient disappearance or gradient explosion. At the same time, the lightweight attention structure SE block is used. The idea of SE block is to start from the weight of spatial information, and use the feature map to obtain the weight of each layer after batch normalization optimization through a series of convolution operations. Firstly, the feature map with input of *H* × *W* × *C* is averaged and pooled into a vector of 1 × 1 × *C*, and then the weight of 1 × 1 × *C* is obtained by two convolutions of 1 × 1. Finally, the feature map of each input channel is multiplied by the vector to obtain the final output.

The activation function used by MobileNetv3 is h-swish, which is obtained by h-sigmoid. It has the characteristics of easier calculation and faster learning, as shown in Equation 1:


(1) $$h-swish=x\frac{ReLU6\left(x+3\right)}{6}$$

In the Mobilenetv3 model, the bottleneck layer is the middle layer between the image feature input and the last fully connected layer, which could retain the feature input. Since the model parameters of MobileNetv3 model have good generalization ability after extensive training of ImageNet data set, the training parameters of this network in ImageNet were transferred.

The input image outputs a feature vector of 1 × 1 × 1280 through the bneck layer and then enters the full connection layer through the dropout layer to reduce the dimension of the feature vector to 1×1×*c*, where *c* represents the number of categories in the data set. When *c*=7, there are seven types of maize. Finally, the output of the full connection layer is processed by Softmax regression to obtain the probability distribution of each class. The calculation formula is:


(2) $$P_{j}=\frac{n_{j}}{\sum_{k=1}^{c}n_{k}}$$

In Equation 2, *p_j_* represents the classification probability of category *j*, *n_j_* represents the output of the full connection layer, and *c* represents the total number of categories in the data set.

We combined the feature extraction of the MobileNetv3 model with the ImageNet data set in the source domain with the fully connected modules, so as to adapt to the classification task and perform the training on the double-side maize image data set to obtain the maize seed network model.

### Improvement of network structure

Based on the above analysis, we know that the MobileNetv3 network model has the characteristics of few parameters and low delay. To solve the problem of low accuracy in current maize classification due to similar morphology, transfer learning can be introduced on the basis of MobileNetv3 network. Based on the characteristics of MobileNetv3 network model, the requirements for device performance can be greatly reduced. In the process of transfer learning, the large-scale shared parameters in the neural network of the pre-training were first migrated, and the initial training weight of the source domain model was transferred to the new network for initialization, so as to obtain the prior knowledge on the large data set. Then, the training was conducted according to the self-built dataset, and the parameters in the model were adjusted through subsequent learning, so as to obtain the classification model. The maize seed classification algorithm combined with transfer learning can not only improve the training efficiency and accuracy of the network model, but also improve the algorithm performance based on the pre-training basis of large data sets. The final model in training will have better generalization ability and robustness.

**Figure 1:**
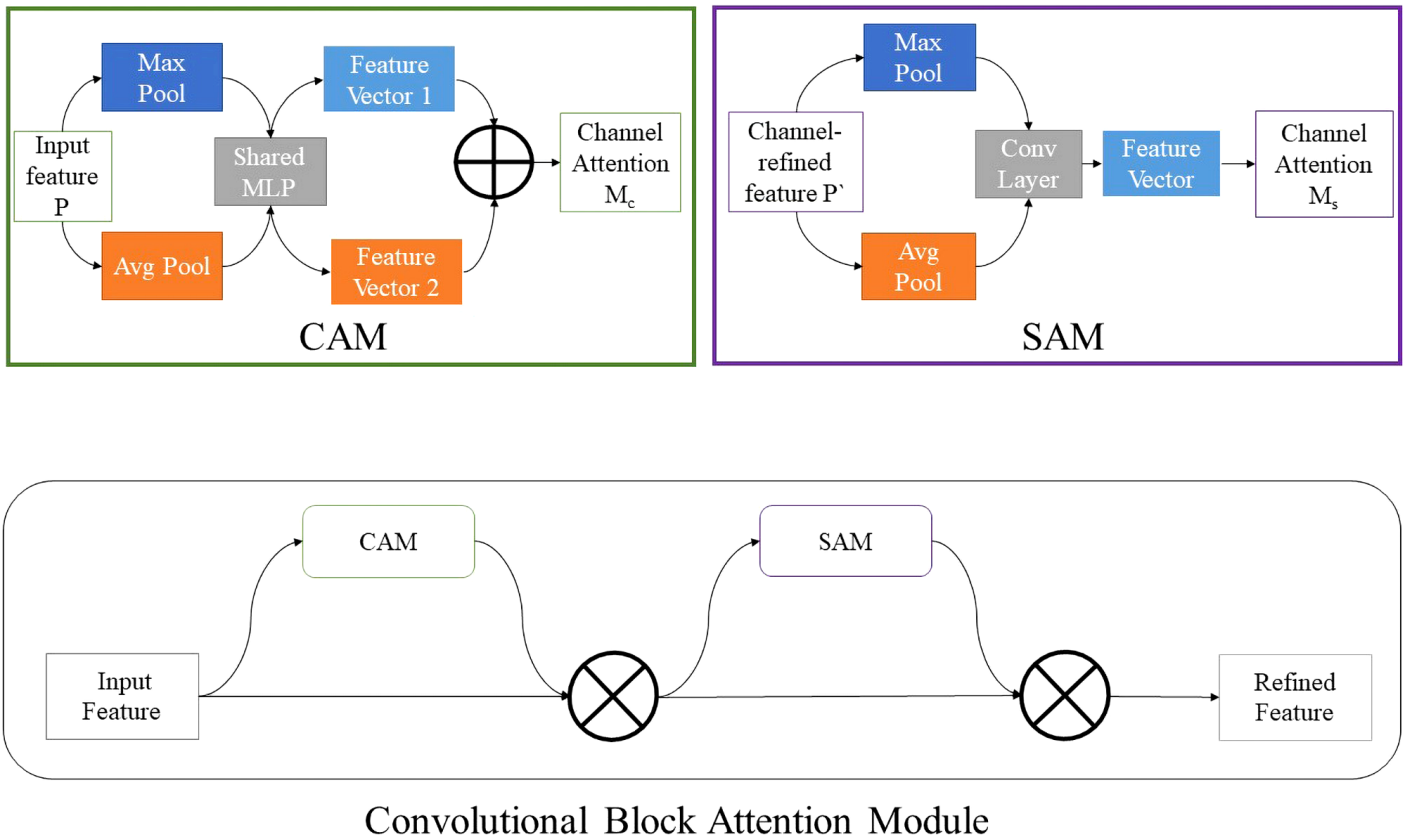
The structure of CBAM. CAM and SAM are included, representing the channel attention mechanism and the spatial attention mechanism respectively.


In addition, the improved MobileNetv3 network introduced CBAM attention mechanism. The structure of CBAM is shown in **Figure 1**. The application of attention mechanisms enabled the neural network to focus on important features and suppress unnecessary features. CBAM module successively induced a one-dimensional channel attention mechanism feature graph *M_c_* and a two-dimensional space attention mechanism feature graph *M_s_*, and the calculation process is shown as follows:

(3) $$P^{'}=M_{c}\left(P\right)⊗P,P^{''}=M_{s}\left(P^{'}\right)⊗P^{'}$$

In Equation 3, *P* is the input image, ⊗ is elements multiplication. In the process of multiplying, the attention mechanism replicates: channel attention mechanisms propagate along spatial dimensions and vice versa. $P^{'}$ is the weighted result of the channel attention mechanism, and $P^{'}$ is the final output obtained. CBAM includes the channel attention mechanism CAM and the spatial attention mechanism SAM. Input the feature map *P* and apply max-pooling and avg-pooling to each spatial position, then it can obtain two *C* × 1 × 1 vectors, which are respectively sent to shared MLP. Finally, the two were combined, and the channel attention *M_c_* was obtained after activation function. The input feature map *P* and apply max-pooling and avg-pooling to each integration to obtain two 1×*H* ×*W* feature vectors, which were then sent to the standard convolution layer according to the channel cost, and *M_s_* was obtained after function activation. Suppose the size of the input image is *C* × *H* × *W*, and *C*, *H* and *W* correspond to the channel, height and weight respectively. Firstly, the spatial information in the feature map was extracted by means of average pooling and maximum pooling, and two different operation results were generated: *P_avg_* and *P_max_*, which represent the feature results generated by average pooling and maximum pooling of input feature map *P*, respectively. Then, the two description results were extracted into the shared network to generate channel attention feature graph *M_c_*. After the shared network was applied to the description of each feature, the output feature vector was obtained by adding elements by elements. The calculation method is as follows:


(4) $$\begin{array}{l} M_{c}=\sigma \left(p_{1}\left(p_{2}(P\right)\right)+p_{1}\left(p_{3}\left(P\right))\right)\\ =\sigma \left(W_{1}\left(W_{o}(P_{avg}\right)\right)+W_{1}\left(W_{o}\left(P_{max}\right))\right) \end{array}$$

In Equation 4, *P* is the input image, *σ* is the nonlinear activation function Sigmoid, *p*_1_ is the forward calculation function, *p*_2_ and *p*_3_ are the average-pooling function and max-pooing function respectively. *W*_0_ and *W*_1_ are the weights of two linear layers. In order to calculate spatial attention, average pooling and maximum pooling were carried out along the channel first, and they were connected together to generate effective feature descriptions, which were represented by *M_s_* (*P*). The channel information of the feature map was extracted through two kinds of operations to generate two two-dimensional feature maps *P_avg_* and *P_max_*, which represent the average pooling feature and the maximum pooling feature respectively. Then the two-dimensional spatial attention feature map was generated by concatenation operation and convolution operation, whose expression is as follows:


(5) $$M_{s}\left(P\right)=\sigma \left(p\left(p_{c}\left(P_{avg,}P_{max}\right)\right)\right)$$

**Figure 2:**
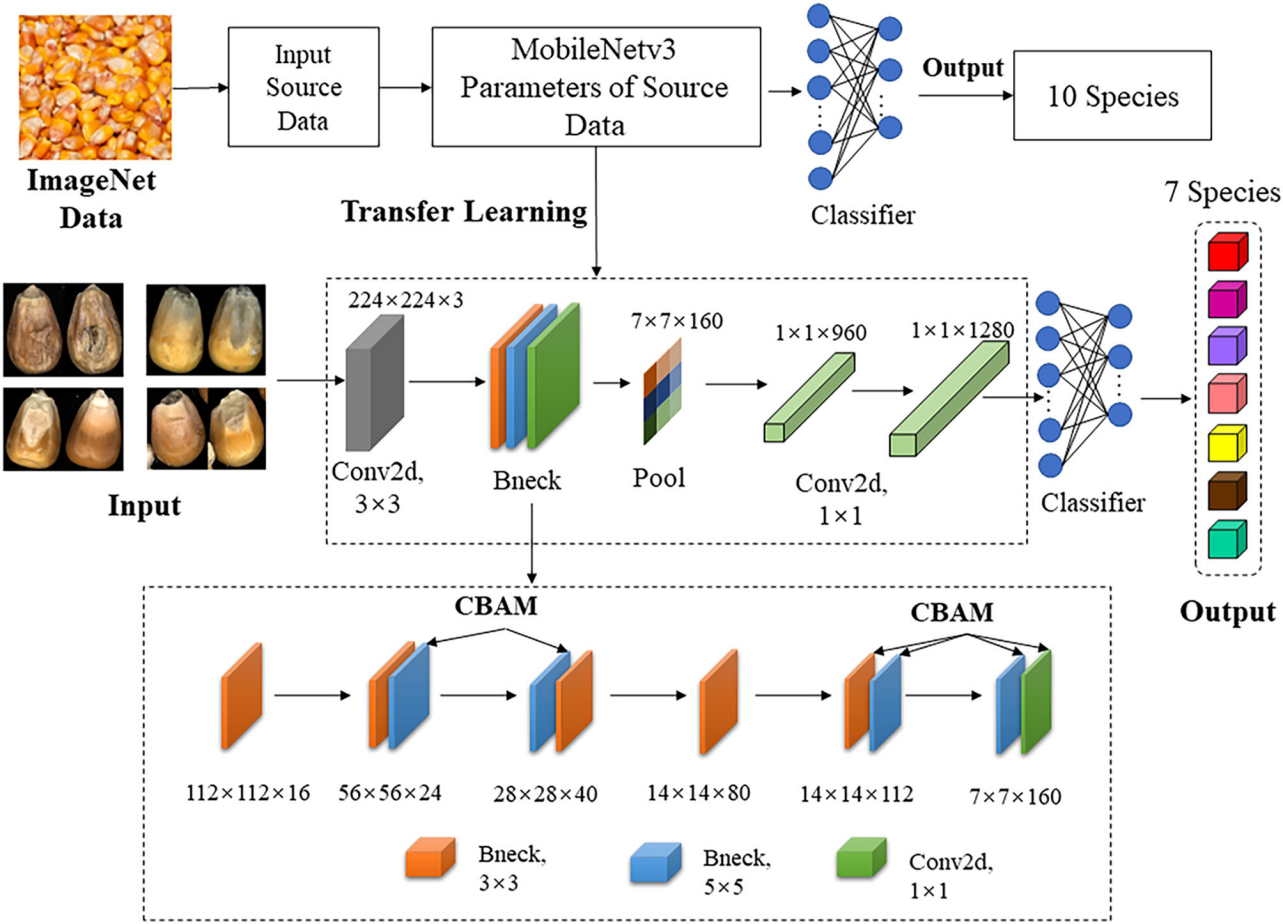
The overview of MobileNetv3-large using CBAM attention mechanism and transfer learning.

In Equation 5, *σ* represents sigmoid activation function, *p_c_* is splicing operation, and *p* is 7×7 convolution operation. The improved MobileNetv3 is shown in **Figure 2**. The structure introduces CBAM attention mechanism to replace the original SE module. The size of each image sample will be resized to the size of 224 × 224 before input.

The Loss Function used in the experiment is the cross entropy, and its expression is shown as follows:


(6) $$L=\frac{1}{N}\sum_{i}L_{i}=-\frac{1}{N}\sum_{i}\sum_{c=1}^{M}y_{ic}\log p_{ic}$$

In Equation 6, *M* is the number of classes, and *y_ic_* is the sign function (0 or 1), if the true class of sample *i* is equal to *c* takes 1, otherwise takes 0. *p_ic_* is the probability that the observed sample *i* belongs to class *c.*

## Experiments

### Datasets

In this experiment, we used the GrainSpace (Fan et al., 2022a) as the dataset for the test model. GrainSpace is a large-scale data set for fine-grained and domain adaptation, which is mainly used for grain recognition. GrainSpace contains three types of grains, namely maize, rice and wheat. We selected the maize image as the data set of this experiment, which is divided into seven categories, and divided into double-sided images and single-sided images.

The maize image is shown in **Table 1**. According to the classification standard formulated by ISO55270 Cereals, maize seeds can be divided into normal and six types of damaged and unsound seeds: fusarium (FM) seeds, sprouted (SD) seeds, mouldy (MY) seeds, broken (BN) seeds, attacked by pests (AP) seeds and heated (HD) seeds. Among these maize seeds, FM, MY, BP maize seeds represent the proportion of maize seeds polluted by Fusarium or fungi; SD, AP and HD maize seeds correspond to the nutritional components of maize. All images are in PNG format. Due to the interference of various factors during the shooting of various maize images, maize grain images with complete shape and standard features are preferred as the data set of the experiment. All the acquired maize images were obtained through cropping. Due to the varying sizes of the maize, the dimensions of each image are not fixed, but the maximum value will not exceed 300 × 400. A total of 12784 training data were obtained. The ratio of train set to validation set was 9:1.

**Table 1:** Part of maize image display.

Class No.	Name	Description	Image(double-side)	Image(single-side)	Train	Validation
1	NOR	Normal	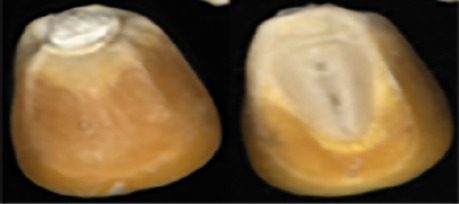	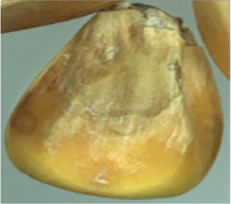	1671	186
2	AP	Attacked by Pests	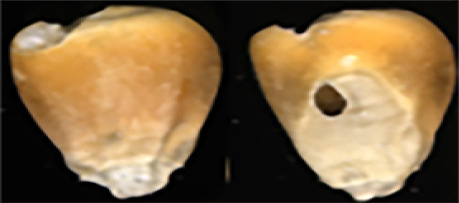	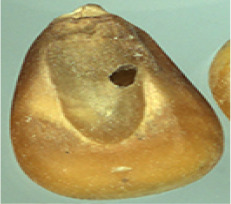	1634	181
3	BN	Broken	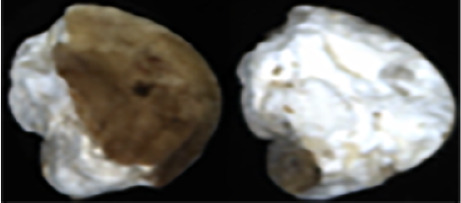	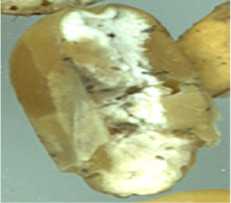	1647	183
4	FM	Fusarium	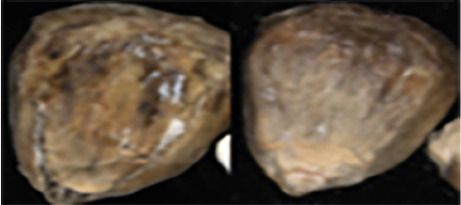	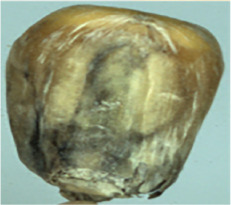	1651	183
5	HD	Heated	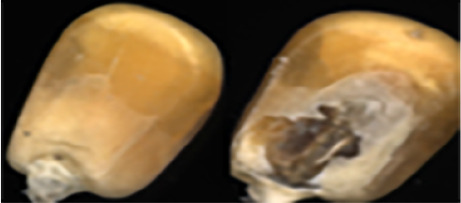	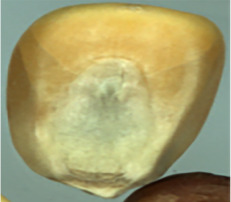	1638	182
6	MY	Mouldy	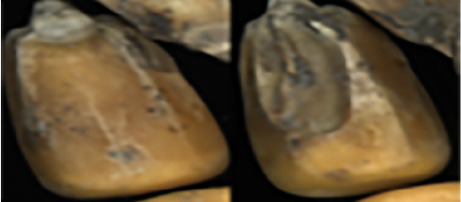	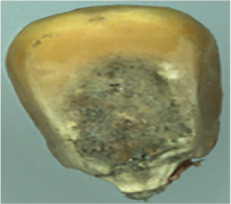	1624	180
7	SD	Sprouted	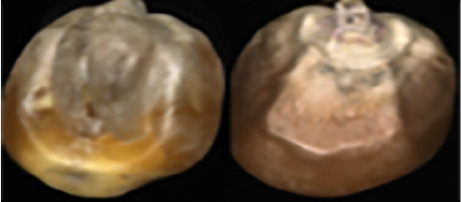	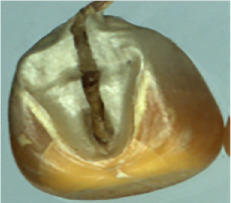	1639	182

### Implementation details

The server platform configuration of the experiment is as follows: CPU is Intel Core I5-10400, 6 core and 12 threads, GPU is NVIDIA GeForce RTX2080ti. The operating system is Windows10 and the Python version is 3.7. The torch version in the deep learning framework is 1.10.0, and the torchvision version is 0.11.0; CUDA version is 11.0; CUDnn version is 8.0. In the experiment, the pre-training weights of MobileNetv3-large models trained on ImageNet were used to initialize the model, and the modified model was used to train the maize seed data set. Adam is used by the model designer, and the Loss Function is cross entropy. Considering the memory and model generalization ability, the learning rate is set to 0.0001. Adam optimization algorithm and h-swish activation function were adopted. Set batchsize to 32 and epochs to 40. Using the Adam optimizer can automatically modify the learning rate with the training process, which can speed up the training speed and improve the performance of the model. We use the h-swish function because it can alleviate the disappearance of the gradient, prevent over-fitting, and accelerate the convergence rate of the gradient descent. Moreover, the computational cost is low, and the computer runs faster.

### Result analysis and visualization

**Figure 3:**
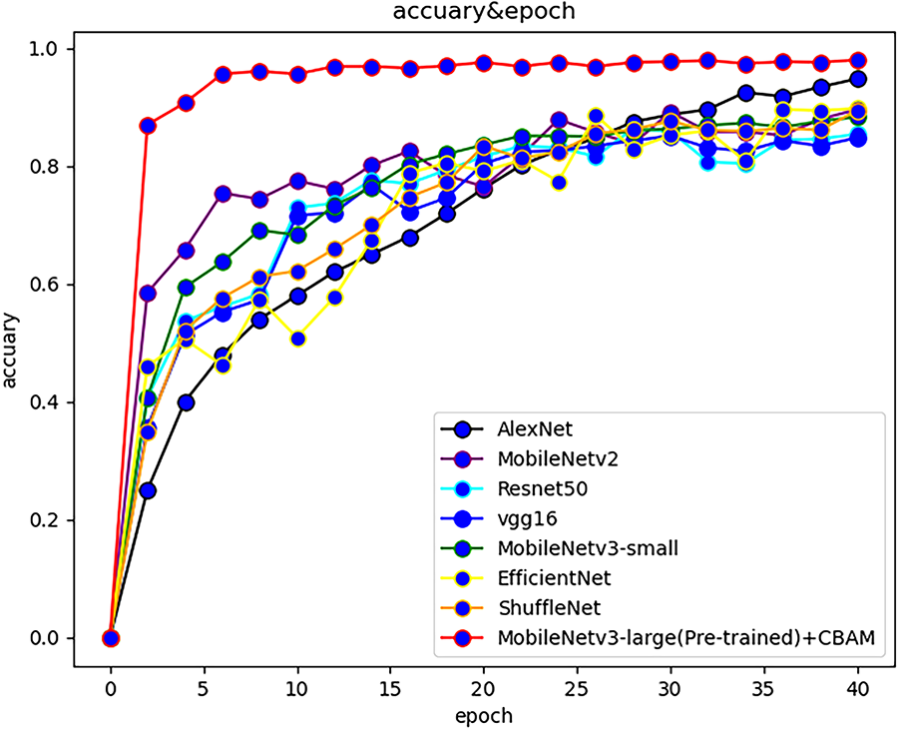
The accuracy of various algorithms varies with the number of epochs.

**Figure 4:**
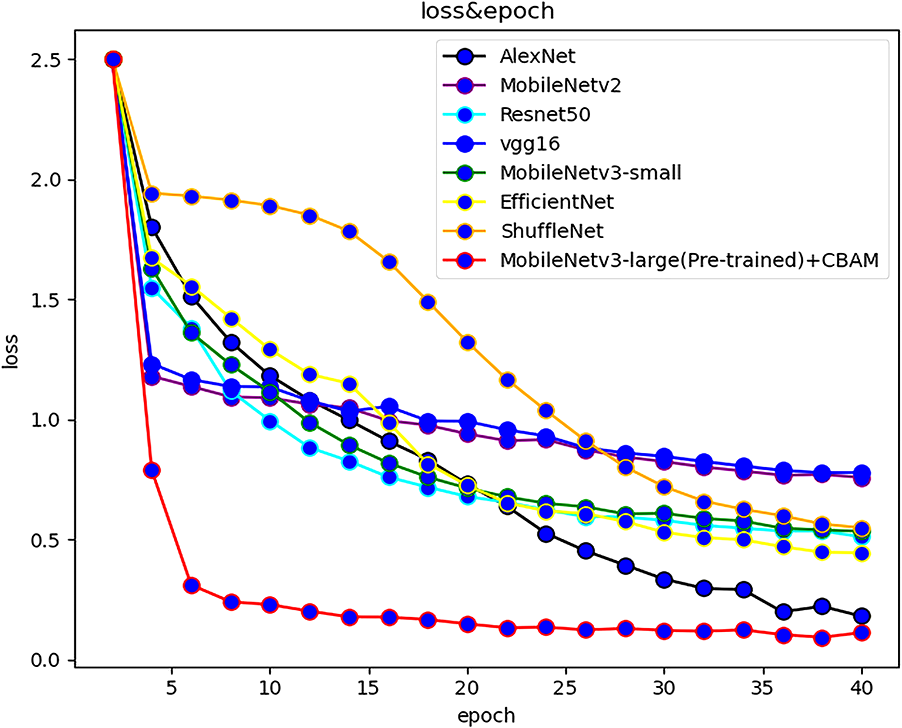
The loss of various algorithms varies with the number of epochs.

At present, there are many convolutional networks applied in deep learning. In order to reflect the effectiveness of the models, popular models such as MobileNetv2, ResNet50 and VGG were selected to compare with the MobileNetv3 network, and the above models were respectively used for training in the self-built dataset of this study. During the training process, the test set and training loss value were recorded for each training cycle completed by the model. The variation of the accuracy rate of each network with the number of epochs is shown in **Figure 3**, and the variation of the loss function with the number of epochs is shown in **Figure 4**.

The accuracy of the improved MobileNetv3 algorithm is more than 10% higher than that of other computational majority methods. The training speed is the fastest, reaching 8.23bit/s, and the test time is only 35.25ms. The reasons are as follows: (1) Depthwise separable convolution was used to reduce the amount of computation and parameters of the model, and the reversed residual network structure constructed was helpful for feature extraction; (2) The CBAM was introduced to integrate the attention mechanism in channel and space, so that the important features are enhanced, while the unimportant features were suppressed; (3) The weight of large data sets in the model can be used to pool network parameters in similar tasks, and it improved the performance of the model. **Table 2** shows the summary of experimental results of each model on the maize seed data set. Based on the aforementioned results, in addition to our method, AlexNet, EfficientNet, and MobileNetv3 - large also demonstrated excellent training outcomes with validation accuracies exceeding 90%. However, ResNet101 and VGG16 exhibited slower training speeds due to their larger model parameters and greater computational requirements, resulting in longer processing times.

**Table 2:** Experimental results of various algorithms.

Model	Prediction (%)							Accuracy (%)	Training speed(bit/s)	Test time (s)
	NOR	AP	BN	FM	HD	MY	SD			
ResNet50	92.80	82.53	97.65	98.14	64.71	73.02	92.24	81.77	2.71	126.15
ResNet101	90.64	81.65	96.51	96.84	63.37	72.53	91.55	78.73	1.43	129.45
VGG16	97.14	91.15	98.33	94.71	66.57	83.52	95.63	86.44	1.33	195.41
AlexNet	82.81	88.14	95.62	96.65	82.20	92.31	93.43	94.80	2.63	128.33
ShuffleNet	80.16	75.67	75.94	96.63	68.14	76.37	79.43	77.24	3.67	50.58
EfficientNet	89.73	96.54	96.83	99.26	70.38	85.59	95.64	89.41	2.64	62.67
EfficientNetv2	90.65	97.65	98.32	99.12	72.23	86.54	96.75	90.90	2.20	46.89
MobileNetv2	96.80	94.96	87.57	97.32	81.36	86.81	97.82	85.26	6.71	43.52
MobileNetv3-small	96.81	94.34	98.96	97.76	84.32	87.93	98.74	87.40	7.82	40.63
MobileNetv3-large	97.24	98.00	97.54	97.74	90.21	96.84	98.85	96.31	8.20	37.64
MobileNetv3-large+CBAM	97.40	98.51	99.03	99.06	91.67	98.49	99.10	97.95	8.23	35.25

A group of maize seed images were selected from other data sets and introduced into the model for classification. The average accuracy of maize seed classification is more than 97%, and the accuracy of some categories of maize seeds classification is more than 99% (such as BN, FM), but the classification accuracy of HD is low. Through analysis, there are two reasons for the decline of HD maize recognition accuracy: Firstly, some maize seeds have complex features, which are highly similar to other maize seeds; Secondly, when the images in the data set were screened, the number of maize seed samples with certain features was small. When CBAM attention mechanism was introduced, the recognition accuracy of image classification by MobileNetv3 algorithm has been improved slightly, and the number of references and the time required for image recognition were reduced.

We selected 50 images for each type of maize seed in other datasets, then observed the prediction results of each type of maize by this method, and drew the confusion matrix. The confusion matrix has five evaluation indexes: *P*, *R*, *F*1, *TPR*, *FPR*. Their calculation is as follows:


(7) $$P=\frac{TP}{TP+FP}$$

(8) $$R=\frac{TP}{TP+FN}$$

(9) $$F1=\frac{2\times P\times R}{P+R}$$

(10) $$TPR=\frac{TP}{TP+FN}$$

(11) $$FPR=\frac{FP}{FP+TN}$$

Where *P* represents precision, *R* represents recall, and *F*1 represents F1-score. *TP* (True Positives) represents the number of true samples correctly classified as positive. *TN* (True Negative) represents the number of true samples incorrectly classified as negative. *FP *(False Positive) represents the number of false samples incorrectly classified as positive. *FN* (False Negative) represents the number of false samples correctly classified as negative. *TPR* (True Positice Rate) represents the proportion of the positive samples of the predicted pair to the total positive samples. The *FPR* (False Positive Rate) represents the proportion of positive samples with wrong prediction to all positive samples.

**Figure 5:**
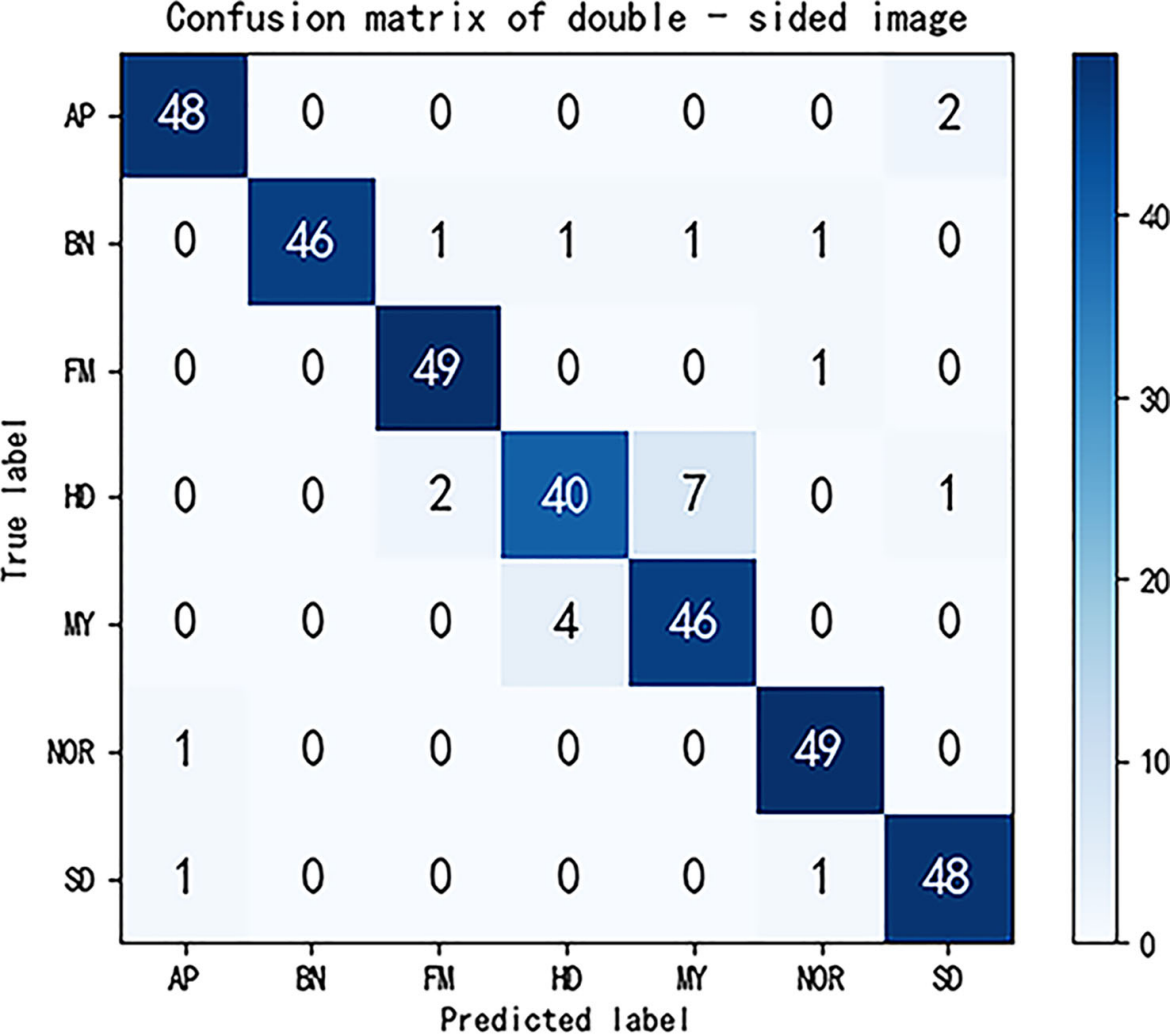
Confusion matrix diagram for double-sided maize seeds forecasts.

The confusion matrix after completion is shown in **Figure 5**. Calculated from the confusion matrix, the accuracy true positive rate of the maize seed image classification model reached 93.14% and the false positive rate reached 1.14%. In addition, the precision, recall rate and F1 values of all kinds of maize seed images were caused by **Table 3**. Compared with previous methods for maize seed detection, the method proposed in this paper not only accurately detects maize affected by defects, but also identifies the types of defects, with a shorter detection time. In conclusion, the method in this paper has high feasibility in the task of maize classification. However, there are certain limitations in our experiment. Firstly, we only conducted detection on 7 types of maize, and when faced with multiple types of defects, we cannot determine the effectiveness of this detection method. Secondly, when detecting maize with highly similar defect types, our method still has issues with category detection errors. Lastly, during the experimental process, we only selected 50 images per category of maize and created a confusion matrix based on the classification results. We then calculated metrics such as accuracy, recall, F1 score for each category of maize, but these test results may contain some degree of error.

**Table 3:** the precision, recall, F1 value of various maize classifications in double-sided images.

Class No.	Class Name	P (%)	R (%)	F1(%)
1	NOR	94.23	98.00	96.08
2	AP	96.00	96.00	96.00
3	BN	100.00	94.00	96.91
4	FM	94.23	98.00	96.08
5	HD	88.88	80.00	84.21
6	MY	85.18	92.00	88.46
7	SD	94.11	96.00	95.05

### Ablation experiment

In the ablation experiment, we use the control variable method to prove the necessity of a module by removing or adding the function of a module. If the performance results obtained after the ablation experiment change significantly, it shows that the module plays a decisive role.

The ablation experiment was divided into four phases. The first step was tested and validated using only the MobileNetv3 algorithm without the use of transfer learning and attention mechanisms. The second step was to use the attention mechanism, but not transfer learning. The third step was to use transfer learning without the attention mechanism. The fourth step was to use both the attention mechanism and transfer learning. In order to verify the influence of attention mechanism, the attention mechanism module was frozen so that it cannot be used. Similarly, this method was also applicable to transfer learning.

**Figure 6:**
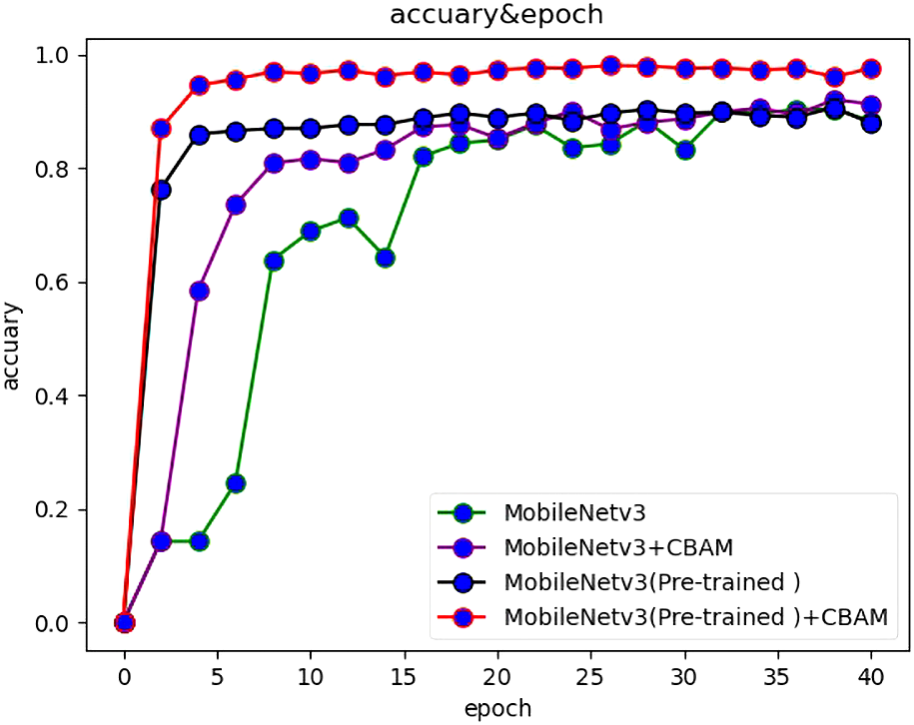
The accuracy of various ablation methods varies with the number of epochs.

**Figure 7:**
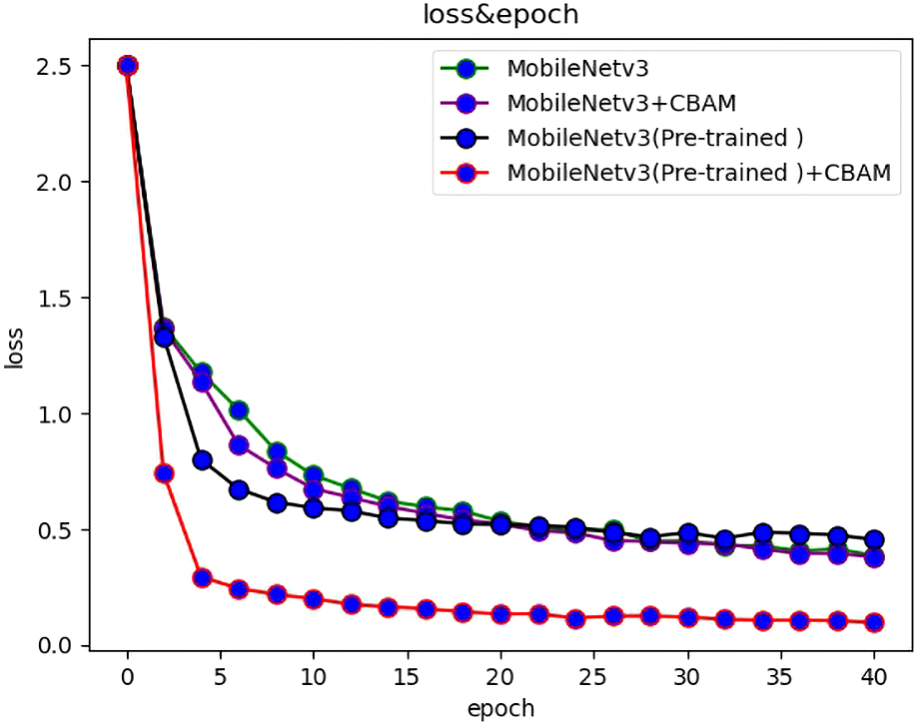
The loss of various ablation methods varies with the number of epochs.

After experimental verification, the accuracy and loss values of each method change with the number of iterations, as shown in **Figures 6**, **7**.

After the use of transfer learning, the recognition accuracy of the model was greatly increased, which increases by 62% when epoch is 1. With the increase of epoch, the gap between the two gradually decreases. When epoch is 20, the gap between the two Narrows to less than 5%; when epoch is 40, the accuracy of the two is almost the same. After the introduction of CBAM attention mechanism, the recognition accuracy of the model can be improved by 7%. In conclusion, for the MobileNetv3 network for double-sided maize seed classification and recognition, the primary factor affecting the accuracy is transfer learning, and the secondary factor is attention mechanism.

**Figure 8:**
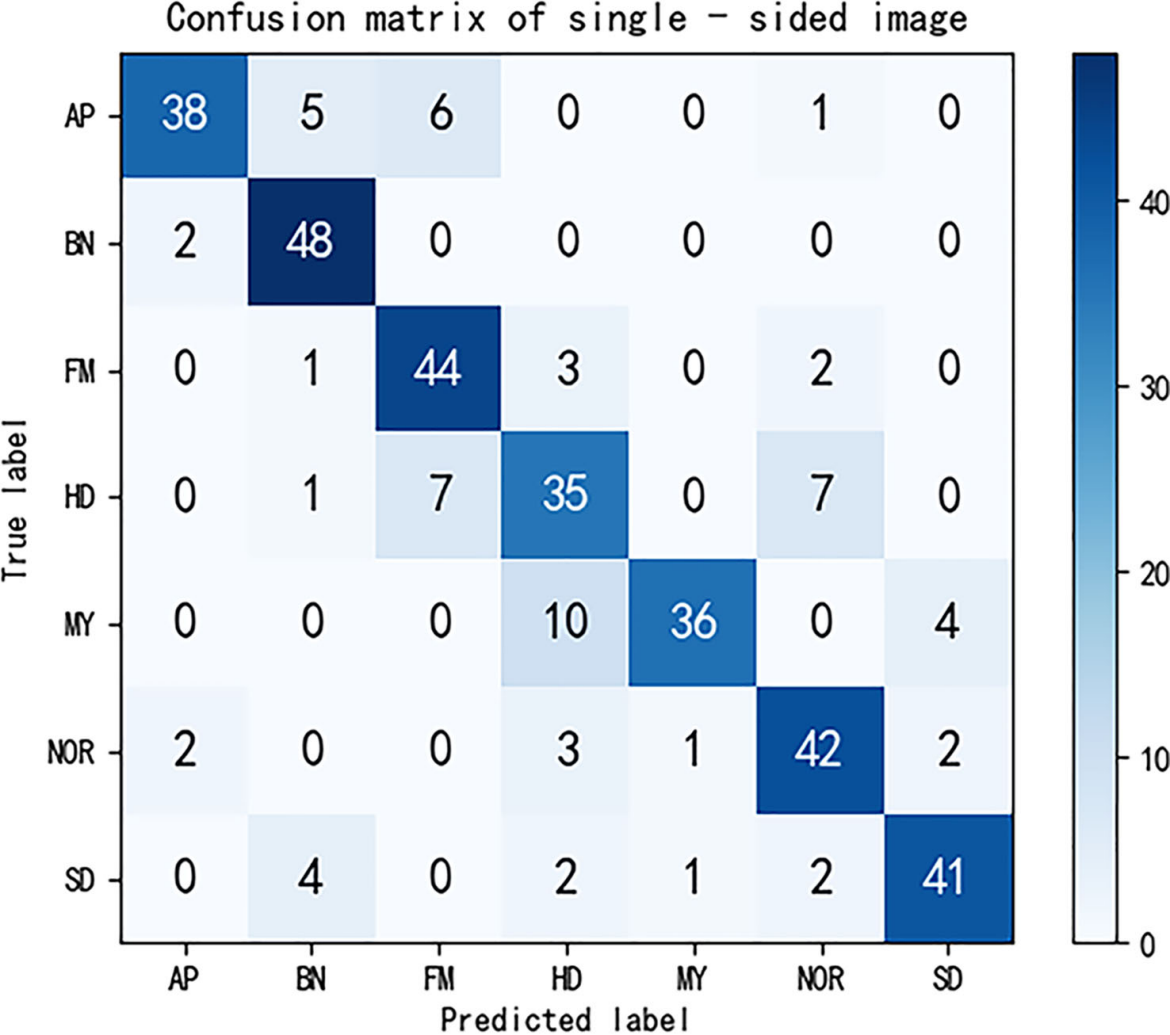
Confusion matrix diagram for single-sided maize seeds forecasts.

At the same time, in order to verify the improvement of the classification accuracy of double-sided maize seed images, a dataset of single-sided maize seed images was used as the control experiment. According to the confusion matrix shown in **Figure 8**, the true positive rate was 81.14% when using single-sided maize seed images, which was 12% lower than that using double-sided maize seed images. The false positive rate was 3.14%, which was 2% higher than that of double-sided maize seed images. The accuracy, recall and F1 values of various types of maize classification calculated according to the confusion matrix are shown in **Table 4**. From this table, it can be analyzed that the overall classification accuracy under one-sided maize seeds is about 15% lower than the overall classification accuracy under double-sided maize seeds.

**Table 4:** The precision, recall, F1 value of various maize classifications in single-sided images.

Class No.	Class Name	P (%)	R (%)	F1 (%)
1	NOR	77.78	84.00	80.77
2	AP	90.48	76.00	82.61
3	BN	81.36	96.00	88.07
4	FM	77.19	88.00	82.24
5	HD	66.04	70.00	67.96
6	MY	94.74	72.00	81.82
7	SD	87.23	82.00	84.54

Through the comparative analysis of the above ablation experiments, it is found that transfer learning has the greatest influence on the accuracy, which can increase the prediction rate by 62% during the first training, and it is the most important factor affecting the accuracy of image recognition. Secondly, the accuracy of double-sided image can be improved by 12% than that of single-sided image. The effect of attention mechanism on image recognition accuracy is 8% higher than without attention mechanism. In summary, the influencing factors were ranked as transfer learning greater than double-sided maize seed images and greater than attention mechanisms.

## Conclusion

The main contribution of this paper is to introduce deep learning into the field of maize seed image classification and recognition, and propose an improved MobileNetv3 algorithm model, achieving a comprehensive classification true positive rate of 93.14%. The average prediction, recall, and F1 score of maize classification reached 93.23%, 93.43%, 93.26% and the average test time is only 35.25 ms. In future research, we will attempt to conduct defect detection studies on other types of grains such as rice and wheat, and explore different methods to achieve superior detection results. Our approach enables automatic classification and detection of maize, thereby identifying higher quality maize and contributing to the efficient allocation of human and material resources, while promoting the advancement of intelligent agricultural production.

## Data availability statement

The raw data supporting the conclusions of this article will be made available by the authors, without undue reservation.

## Author contributions

CL was responsible for the conception and design of research, the design of experimental methods, and the review and revision of papers. ZC was responsible for software development and design, experimental design verification and verification, experimental data analysis, and writing the first draft of the paper. WJ was responsible for supervision and guidance of research projects, review and revision of papers. YZ was responsible for the conception and design of research, the acquisition of research funds, the collection of research resources, the supervision and guidance of research topics, and the review and revision of papers. XW was responsible for data collation and management, research project management, paper review and revision. All authors contributed to the article and approved the submitted version.
